# Septic patients without obvious signs of infection at baseline are more likely to die in the ICU

**DOI:** 10.1186/s12879-022-07210-y

**Published:** 2022-03-02

**Authors:** Francesco Campanelli, Agnès Soudry-Faure, Aurélie Avondo, Jean-Baptiste Roudaut, Jean-Pierre Quenot, Patrick Ray, Pierre-Emmanuel Charles

**Affiliations:** 1 Centre Régional Universitaire Des Urgences, Hôpital F. Mitterrand, C.H.U. DIJON, Bd Mal de Lattre de Tassigny, Dijon, France; 2 Centre d’Investigation Clinique, Hôpital F. Mitterrand, C.H.U. Dijon, 14 rue Gaffarel, Dijon, France; 3 Service de Médecine Intensive Réanimation, Hôpital F. Mitterrand, C.H.U. DIJON, 14 rue Gaffarel, B.P. 77908-21079 Dijon Cedex, France; 4 Laboratoire Lipness, U.M.R. 1231, INSERM, Université de Bourgogne-Franche Comté, 7 Bd Jeanne d’Arc, Dijon, France; 5 INSERM, CIC 1432, Module Épidémiologie Clinique, Université de Bourgogne-Franche Comté, Dijon, France

**Keywords:** Sepsis, Infection, Emergency department, Intensive Care Unit, Time-to-antibiotics, Fever

## Abstract

**Objective:**

Early identification of sepsis is mandatory. However, clinical presentation is sometimes misleading given the lack of infection signs. The objective of the study was to evaluate the impact on the 28-day mortality of the so-called “vague” presentation of sepsis.

**Design:**

Single centre retrospective observational study.

**Setting:**

One teaching hospital Intensive Care Unit.

**Subjects:**

All the patients who presented at the Emergency Department (ED) and were thereafter admitted to the Intensive Care Unit (ICU) with a final diagnosis of sepsis were included in this retrospective observational three-year study. They were classified as having exhibited either “vague” or explicit presentation at the ED according to previously suggested criteria. Baseline characteristics, infection main features and sepsis management were compared. The impact of a vague presentation on 28-day mortality was then evaluated.

**Interventions:**

None.

**Measurements and main results:**

Among the 348 included patients, 103 (29.6%) had a vague sepsis presentation. Underlying chronic diseases were more likely in those patients [e.g., peripheral arterial occlusive disease: adjusted odd ratio (aOR) = 2.01, (1.08–3.77) 95% confidence interval (CI); *p* = 0.028], but organ failure was less likely at the ED [SOFA score value: 4.7 (3.2) vs. 5.2 (3.1), *p* = 0.09]. In contrast, 28-day mortality was higher in the vague presentation group (40.8% vs. 26.9%, *p* = 0.011), along with longer time-to-diagnosis [18 (31) vs. 4 (11) h, p < 0.001], time-to-antibiotics [20 (32) vs. 7 (12) h, p < 0.001] and time to ICU admission [71 (159) vs. 24 (69) h, p < 0.001]. Whatever, such a vague presentation independently predicted 28-day mortality [aOR = 2.14 (1.24–3.68) 95% CI; *p* = 0.006].

**Conclusions:**

Almost one third of septic patient requiring ICU had a vague presentation at the ED. Despite an apparent lower level of severity when initially assessed, those patients had an increased risk of mortality that could not be fully explained by delayed diagnosis and management of sepsis.

**Supplementary Information:**

The online version contains supplementary material available at 10.1186/s12879-022-07210-y.

## Introduction

Sepsis is a life-threatening organ dysfunction caused by a dysregulated host response to infection, leading to a risk of death ranging from 15 to 40%, if septic shock occurred [1, 2]. Given the disease burden worldwide and its high mortality rate, efforts have been done in order to improve sepsis outcome [3–5].

Accordingly, early diagnosis and therapy including appropriate antibiotics and fluid administration within the first hour are the cornerstone of sepsis management [6].

However, suspecting sepsis is challenging since it relies on the diagnosis of infection together with organ failure assessment through the quick Sepsis-related Organ Failure Assessment (qSOFA) score calculation. Although qSOFA implementation is part of the Surviving Sepsis Campaign (SSC) guidelines, its screening value has been repeatedly questioned, given its low negative predictive value [7, 8].

Most of all, sepsis recognition could be delayed if presenting symptoms, are not clearly and immediately suggestive of infection (i.e. the so-called vague presentation), especially upon admission in the Emergency Department (ED), leading in turn to late appropriate therapy. However, little is known about vague symptoms frequency in patients finally diagnosed with sepsis. In addition, whenever clinical presentation of infection is predictive of patient outcome remains to be more extensively evaluated. In a retrospective cohort study, Filbin et al. found that about one third of septic patients presented to the ED with vague symptoms [9]. Moreover, in-hospital mortality was significantly higher in such patients if compared with those in whom infection was obvious. Similarly, other authors have reported that normothermia was not infrequent in septic patients and was associated with a poorer outcome as compared with fever [10–12]. However, published data are scarce and the reasons why clinical initial presentation influences the outcome remain not fully understood, since the lack of prompt and adequate management of sepsis in the patients with vague symptoms did not necessarily account for the whole difference of survival reported so far.

We hypothesized therefore that host related factors could also explain such a gap.

The main objective of the study was to compare in terms of outcome the patients presenting with the so-called “vague” presentation of sepsis, to those harbouring explicit signs and symptoms upon ED admission. We conducted therefore a retrospective observational study in a cohort of patients with a final diagnosis of sepsis, evaluated in the ED before Intensive Care Unit (ICU) admission.

## Materials and methods

### Study design

A retrospective monocentric cohort study was conducted from January 1, 2016, to December 31, 2019. All consecutive adult patients (≥ 18 years old) who presented at the ED of the Centre Hospitalier Universitaire de Dijon (CHU) and were then hospitalized in the Medical Intensive Care Unit (ICU), directly or not, were considered for inclusion.

### Ethical statements

The present study has been conducted in accordance with the declaration of Helsinki. Given its retrospective design, the requirement for informed consent was waived in accordance with the French law on retrospective studies of anonymized data. The institutional review board (Comité de Protection des Personnes Est I, Dijon) has approved the protocol, and the fact that the need for informed consent was waived.

### Inclusion and exclusion criteria

The following inclusion criteria were applied: (i) at least one diagnosis code for any kind of infection according to the International Classification of Diseases, 10th edition (ICD-10); (ii) the presence of sepsis or septic shock criteria according to the Sepsis-3 experts’ panel at any time between ED admission and transfer to the ICU [1].

Patients who had been treated for infection before ED arrival were excluded as well as those who were primarily hospitalized for a non-septic reason and who secondarily (i.e., beyond 48-h following ED admission) met sepsis criteria, then considered as related to an hospital-acquired infection.

### Definition of explicit and vague presentation

Presenting symptoms were collected from triage and/or physician/resident doctor’s notes at the ED. We defined patient presentation as “explicit” according to the criteria used by Filbin et al. and detailed below, since it was considered to rapidly lean the clinician to consider infection [9]. Thus, the so-called explicit symptoms for infection were: hyperthermia or hypothermia (body temperature ≥ 38.5 °C or < 36 °C, respectively), chills, systolic arterial pressure (SAP) ≤ 90 mmHg or mean arterial pressure (MAP) ≤ 65 mmHg, cough with productive sputum, dysuria, reported skin redness or concern for soft-tissue infection, referral for specific diagnosis of infection.

In contrast, patient presentation was considered as vague, if symptoms at the ED did not include any of the explicit symptoms listed above.

### Data collection

We obtained all the data from the electronic medical record system of the hospital. Patient baseline characteristics and past medical conditions were listed. Vital parameters at the ED arrival were collected, and the “quick” Sequential Organ Failure Assessment (qSOFA) score was calculated. Day 1 SOFA score was calculated upon infection was suspected according to current sepsis definitions. Time-to-infection suspicion, time-to-antibiotic administration and time-to-ICU hospitalization were calculated from the first medical contact since it has been automatically recorded within the ED medical chart. First-line antibiotic treatment was considered appropriate according to the available susceptibility testing data of the involved identified pathogen(s), if any. Otherwise, compliance with the current guidelines was considered.

### Outcomes

The primary outcome was all cause 28-day mortality.

The secondary outcomes were in-hospital all-cause mortality, overall ICU length of stay (LOS) and hospital LOS.

### Statistical analysis

In a first set of analysis, patients with vague presentation were compared with those without.

Categorical variables were compared with the chi-square test and the Mann–Whitney test was used to compare continuous variables. In an attempt to identify covariates likely to be independently associated with vague presentation, a multivariate analysis based on a logistic regression model was conducted. Covariates were selected if the p value was less than 0.2 by univariate analysis or if it was considered as clinically relevant.

In a second set of analysis, outcomes including 28-day mortality were assessed. We hypothesized that the 28-day mortality rate would rise from 15 to 30% in patients with vague symptoms as compared to those with explicit sepsis [9]. We then calculated a sample size of 348 patients in order to reach a statistical power of 0.80.

The 28-day survival was then analysed through the corresponding Kaplan–Meier curves construction (i.e., vague vs. explicit), which were compared with the log-rank test. Potential explanatory variables were then assessed through univariate analysis as described above. Likewise, independent predictors for 28-day mortality were sought through a regression logistic model construction.

Outliers if any were kept in all analysis. No missing data were recorded.

A *p* value of less than or equal to 0.05 was used as the cut-off for all tests of statistical significance.

The JASP software version 0.13.1 was used for all analysis.

## Results

### Study population

**Fig. 1:**
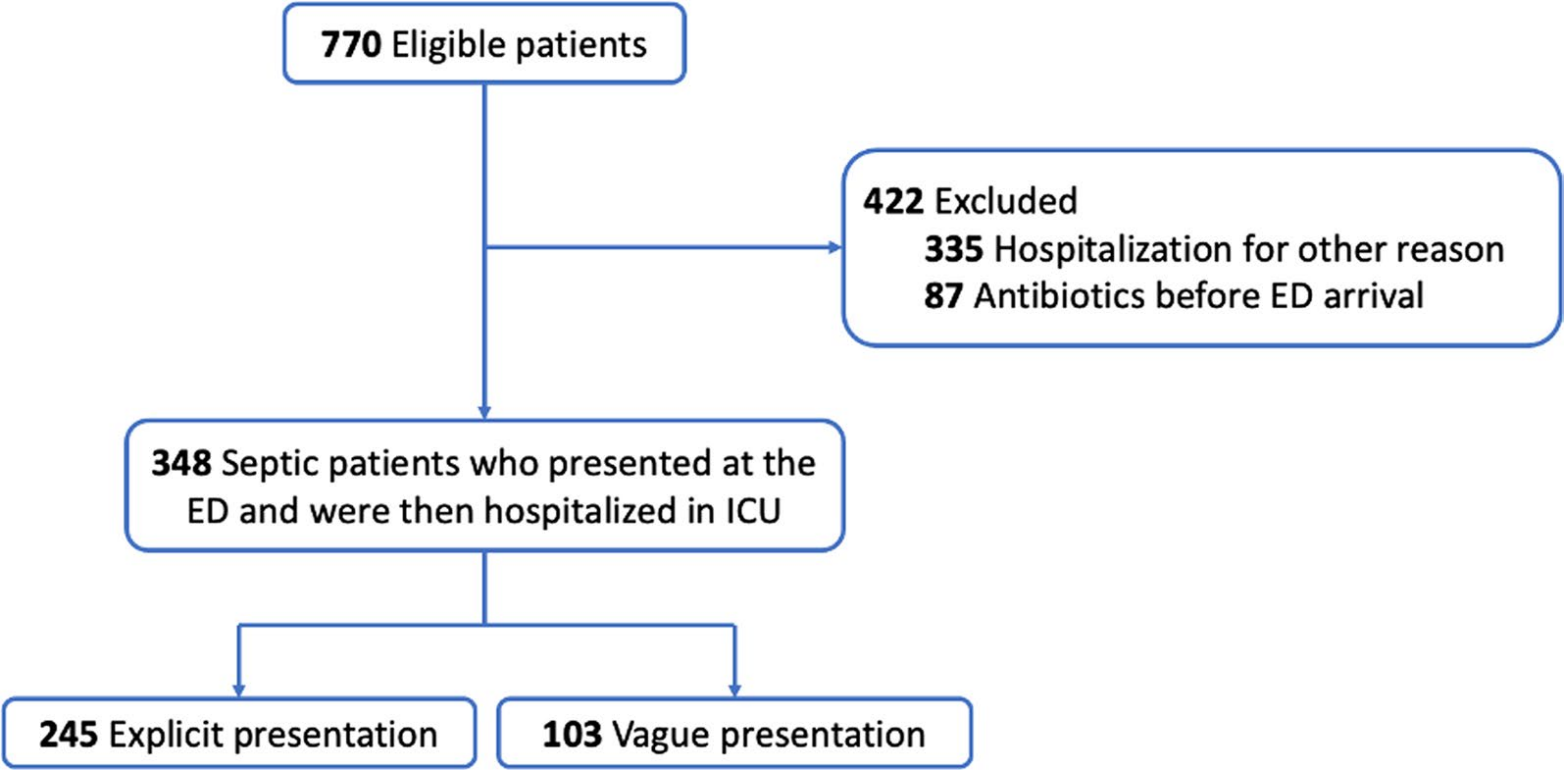
Flowchart of the study. ED: Emergency Department, ICU: Intensive Care Unit

From January 1st, 2016, to December 31, 2019, 770 patients who presented at the ED and were then hospitalized in the ICU had a sepsis diagnosis recorded at the end of their stay (see Fig. 1). Four hundred and forty-two (55%) were excluded from our study since 335 (43.5%) were initially hospitalized for another reason than sepsis or septic shock, and 87 (11.5%) had an antibiotic treatment before arriving at the ED. Finally, 348 patients were included in our study. Of them, 245 (70%) had an explicit presentation for sepsis, and 103 (30%) had a vague presentation.

Baseline characteristics differed between groups regarding underlying diseases since peripheral arterial occlusive disease (PAOD) and chronic kidney disease (CKD) were more likely in the patients with vague presentation of sepsis, whereas asthma and haematological malignancies were more frequently encountered in the other group (Table 1) (Additional files 2; 4; 5).

**Table 1:** Baseline characteristics of patients according to the clinical presentation at the emergency department

Total, n = 348	Explicit n = 245	Vague n = 103	OR	95% CI	*P*
Demographics					
Age, median year (IQR)	70.6 (50–90)	71.1 (52–90)	1.00	0.98–1.02	0.72
Gender female (%)	98 (40)	32 (31)	0.68	0.41–1.10	0.12
Smoke (%)	75 (30.6)	34 (33)	1.12	0.68–1.83	0.66
Alcohol (%)	47 (19.2)	28 (27,2)	1.57	0.92–2.69	0.09
Underlying diseases					
Hypertension (%)	121 (49.4)	59 (57.3)	1.37	0.86–2.19	0.18
CHF (%)	47 (19.2)	19 (18.4)	0.95	0.53–1.72	0.87
Myocardial infarction (%)	49 (20)	24 (23.3)	1.22	0.69–2.11	0.49
Atrial fibrillation (%)	77 (31.4)	35 (34)	1.12	0.69–1.83	0.64
PAOD (%)	33 (13.5)	24 (23.3)	1.95	0.09–3.51	0.025
DVT (%)	27 (11)	10 (9.7)	1.95	1.09–3.51	0.72
Diabetes mellitus (%)	76 (31)	29 (28.2)	0.87	0.53–1.45	0.59
COPD (%)	34 (13.9)	18 (17.5)	1.31	0.70–2.45	0.39
Asthma (%)	17 (6.9)	1 (1)	0.13	0.02–1.00	0.05
CKD (%)	36 (14.7)	19 (18.5)	1.31	0.71–2.42	0.38
Stroke (%)	19 (7.8)	14 (13.6)	1.87	0.89–3.89	0.09
Neurocognitive disease (%)	18 (7.3)	10 (9.7)	1.35	0.60–3-05	0.46
Cirrhosis (%)	18 (7.3)	6 (5.8)	0.78	0.30–2.03	0.61
Immunosuppressive drugs (%)	43 (17.6)	12 (11.6)	0.62	0.31–1.23	0.17
Haematological malignancy (%)	37 (15.1)	7 (6.8)	0.41	0.18–0.95	0.03
Solid cancer (%)	56 (22.9)	25 (24.3)	1.08	0.63–1.86	0.78
Infection site					
Pneumonia (%)	117 (47.8)	52 (50.5)	1.12	0.70–1.77	0.64
Urinary tract infection (%)	53 (21.6)	14 (13.6)	0.57	0.30–1.08	0.08
SST infection (%)	16 (6.5)	4 (3.9)	0.58	0.19–1.77	0.34
GI source of infection (%)	14 (5.7)	6 (5.8)	1.02	0.38–2.73	0.97
Biliary source of infection (%)	7 (2.9)	10 (9.7)	3.66	1.35–9.89	0.01
Bacteremia (%)	91 (37.1)	26 (25.2)	0.57	0.34–0.96	0.03

ED, Emergency Department; OR, Odds Ratio; CI, Confidence Interval; CHF, Chronic Heart Failure; PAOD, Peripheral Arterial Occlusive Disease; DVT, Deep Venous Thrombosis; COPD, Chronic Obstructive Pulmonary Disease; CKD, Chronic Kidney Disease; SST, Skin and Soft Tissues; GI, Gastrointestinal; IQR, Inter Quartile Range

Upon ED admission, patients with vague presentation had higher blood pressure levels, lower core temperature and lower qSOFA value [0.8 (0.8) vs. 1.3 (0.8), p < 0.001]. Although the difference did not reach statistical significance, mean SOFA score at the time of infection diagnosis was also found to be lower if clinical presentation was vague (4.7 [3.2] in the implicit group vs. 5.2 [3.1] in the explicit group, p = 0.09) (Additional file 3: Table S1).

We sought then which characteristics could be independently associated with the vague presentation of sepsis (Table 2). Thus, underlying haematological malignancies was less likely in those patients, as well as bacteremia occurrence and asthma. PAOD was independently associated with a vague presentation. In contrast, sources of infection were similar between the two groups, despite a slightly greater prevalence of biliary infections in the implicit presentation group.

**Table 2:** Independent predictors for vague presentation at the emergency department

Covariate	aOR	95% CI	*P*
PAOD	2.01	1.08–3.77	0.028
Asthma	0.13	0.02–0.97	0.046
Haematological malignancy	0.34	0.14–0.83	0.018
Biliary source of infection	3.99	0.29–0.84	0.009
Bacteremia	0.49	0.29–0.84	0.010

PAOD, Peripheral Arterial Occlusive Disease; aOR, adjusted Odds Ratio; CI, Confidence Interval

### Patients’ management at the ED

As expected, the diagnosis of sepsis was more frequently achieved upon the ED in the explicit presentation group (86.1% vs. 62.1%, p < 0.001) (Table 3). Time-to-diagnosis was longer in the patients with vague presentation [median delay [IQR], 6 (14) vs. 3 (4) h, p < 0.001], as well as the time-to-antibiotic administration [7 (20) vs. 4 (4) h, p < 0.001]. Similarly, the time elapsed between ED and ICU admission was longer in the patients with vague presentation than in others [19 (40) vs. 7 (11) h, p < 0.001], along with a lower rate of ICU direct admission.

**Table 3:** Patients’ management at the emergency department and outcomes according to the clinical presentation of sepsis

Total, n = 348	Explicit n = 245	Vague n = 103	*p*
Sepsis management			
ED wait, mean minutes (SD)	27 (47)	11 (50)	0.82
Sepsis diagnosis upon the ED (%)	211 (86.1)	64 (62.1)	< 0.001
Time to sepsis diagnosis, median hours (IQR)	3 (4)	6 (14)	< 0.001
Time to antibiotics, median hours (IQR)	4 (4)	7 (20)	< 0.001
Time to ICU admission, median hours (IQR)	7 (11)	19 (40)	< 0.001
Direct ICU hospitalisation (%)	192 (78.4)	48 (46.6)	< 0.001
Appropriate antibiotics (%)	131 (53.5)	46 (44.6)	0.13
Outcomes			
28-day Mortality (%)	66 (26.9)	42 (40.8)	0.011
In-hospital Mortality (%)	69 (28.2)	47 (45.6)	0.002
ICU LOS, median days (IQR)	3 (5)	4 (5)	0.24
Overall LOS, median days (IQR)	13 (15)	11 (17)	0.63

ICU, Intensive Care Unit; ARDS, Acute Respiratory Distress Syndrome; GCS, Glasgow Coma Score; LOS, Length of Stay; IQR, Inter Quartile Range

Overall, empirical antibiotics were adequate in 177/348 patients (50.9%), since susceptibility data were not available for 78/348 patients (22.4%). Interestingly, antibiotics were less frequently appropriate in the patients with vague presentation (44.6%) than in others (53.6%), but the difference was not significant.

### Patients’ outcomes

Patients from the vague presentation group had a significantly higher 28-day cumulative risk of death than those who presented with explicit signs of sepsis (40.8% vs. 26.9%, respectively; p = 0.011) (Table 3). Similarly, the Kaplan–Meier survival analysis showed a significative difference between the two groups (p = 0.016) (see Additional file 1: Figure S1).

After adjustment for potential confounders, vague presentation of sepsis remained significantly associated with 28-day mortality (Table 4). In contrast, the ICU LOS as well as the overall hospital LOS was similar (Table 3).

**Table 4:** Independent predictors of all cause death at day-28

Covariate	aOR	95% CI	*p*
Vague presentation	2.18	1.25–3.78	0.006
Age	1.04	1.02–1.06	< 0.001
Arrival body temperature	0.80	0.68–0.94	0.006
Overall SOFA score at diagnosis	1.20	1.10–1.30	< 0.001
Time-to-antibiotics	1.00	0.99–1.00	0.712

aOR, adjusted Odds Ratio; CI, Confidence Interval; SOFA, Sequential Organ Failure Assessment

## Discussion

We show herein that vague presentation is common in septic patients in the ED, since it was found in about 30% of them. Moreover, and strikingly, the absence of explicit symptoms of infection was associated with a poorer outcome despite an apparently lower level of clinical severity in terms of organ failure.

Our findings are in line with previously published ones. Thus, among 654 septic ED patients in one single center from the United States (US), 37% of them exhibited vague presentation [9]. Similarly, in a Swedish cohort including more than 2000 patients, it was reported that 30% of them presented with neither fever nor hypothermia, in accordance with US data, thus delaying sepsis recognition and management [12, 13].

These results suggest that vague presentation including frequent normothermia, could reflect differences regarding the inflammatory response features and magnitude. Interestingly, we show herein that a vague presentation was less likely in patients with hematological malignancies, as well as in those with bacteremia. Altogether, these findings suggest that a low bacterial inoculum could account for the paucity of signs of infection. One could also speculate that the host immune response had been mitigated when clinical presentation of sepsis was vague, as compared to the one encountered in patients with much more explicit symptoms. Unravelling the inflammatory response patterns through key mediators’ measurements would be thereby of great interest in order to find out the molecular basis of these clinical findings. As reported previously, we show herein that a vague presentation was independently associated with mortality. Since immunoparalysis frequently complicates sepsis, thereby compromising the patients ‘outcome, although speculative, one could hypothesize that the lack of infection signs and symptoms reflects such a depressed immune response [14, 15].

Accordingly, cumulative data suggest that there is a strong link between sepsis clinical and biological features, and outcome [16, 17]. Thus, Seymour et al. recently identified four distinct sepsis phenotypes with various risk of mortality as well as treatment responsiveness [18].

Similarly, genomic and transcriptomic data have emphasized to which extent survival could be tightly related to some gene’s expression patterns [16, 19]. To determine to which extent the vague presentation of sepsis could be correlated with peculiar patterns of the host immune response deserves further studies.

More research is needed in order to address this issue, but other explanatory hypothesis should be raised. Basically, delayed sepsis recognition could account for the higher 28-day mortality rate reported in the implicit group, as compared to the patients with obvious signs of infection, since it occurred 14 h earlier in the latter. Actually, and expectedly, antibiotics administration as well as transfer toward the ICU were also achieved significantly later in the vague presentation group. Given the known impact of any delay in sepsis management, especially the door-to-needle time as far as antibiotics are concerned, this could account for the poorer prognosis of the patients in whom the diagnosis of infection is tough [20–22]. However, vague presentation remains associated with a poor outcome even after adjustment for these factors, suggesting that the lack of symptoms could be involved by it-self, thus confirming previously published data [9]. As expected, age, SOFA score value and body temperature were also independent risk factors for death [23–26].

Interestingly, initial empirical antibiotic treatment tended to be more frequently adequate in the explicit group (53.6% vs. 44.6%), although this difference was not significant. Maybe, this could be explained by an easier infection source identification thanks to the collection of more explicit symptoms.

Patients without fever are less likely to be suspected of infection than others. As expected, hyperthermia was infrequent in the included patients with vague presentation. In addition, we found a correlation between body temperature at ED arrival and 28-day mortality. These results are consistent with the findings of Tiruvoipati et al., who have reported that hypothermia in the first 24 h of presentation is associated with higher in-hospital mortality [27]. Accordingly, Young et al. have shown that an elevated peak temperature in the first 24 h in ICU is associated with a decreased in-hospital mortality [11]. Moreover, Kushimoto et al. suggested that the addition of hypothermia to the calculation of the qSOFA score could improve its ability to predict mortality [28]. Finally, it is worth noting that both respiratory and heart rates were similar regardless of vague presentation, whereas qSOFA value reached greater values in the patients with implicit presentation of sepsis. This suggests that previously stated Sepsis-2 criteria could have been more accurate than the latest ones in our population.

However, this study has several limitations. Firstly, given its retrospective design, some patient’s data were sometimes missing or lack of accuracy. Thus, we cannot exclude that some patients have been wrongly classified into the vague presentation group, if some infectious signs present upon ED admission were not mentioned within the medical chart. Similarly, neither imaging nor biological data were considered despite their potential contribution to the sepsis diagnosis. However, clinical assessment, remains the very first line of evaluation of every patient presenting to the emergency room. Secondly, it was a monocentric study. Differences in terms of population characteristics, local epidemiology and provided care may thus exist. As a result, it could be hazardous to translate our findings to another population. In addition, the chosen definition for vague presentation of sepsis could be considered as a matter of concern since it did not include all the potential clinical signs or symptoms likely to suggest the diagnosis of infection. Finally, the only patients admitted to the ICU were considered for inclusion. As a result, those with do not resuscitate order were excluded, thereby limiting our findings to selected patients.

## Conclusions

Almost one third of septic patient requiring ICU admission had a vague presentation at the ED. Despite an apparent less severity initially, such a vague presentation of sepsis was associated with a significantly higher 28-day mortality rate, independently from delayed diagnosis and management. Although further studies are needed, our findings are in accordance with previously published data and provide new insights into this topic. Finding out the immunological and molecular basis of the vague presentation of sepsis deserves future investigations. This could be helpful for designing new and personalized therapeutical approaches of sepsis according to the clinical presentation.

## Supplementary Information

**Additional file 1: Figure S1. **28-day cumulative survival according to the sepsis presentation (i.e., Implicit *vs*. Vague) at the emergency department in 348 critically ill patients diagnosed with sepsis.

**Additional file 2: Figure S2. **Probability of 28-day death according to Body Temperature (BT) measured at the ED in 348 ICU patients diagnosed with sepsis.

**Additional file 3: Table S1. **Severity criteria of infection according to the clinical presentation of sepsis at the emergency department.

**Additional file 4: Table S2. **Confirmed pathogen according to the clinical presentation of sepsis at the emergency department.

**Additional file 5: Table S3. **Clinical predictors of all cause death at day-28.

## Data Availability

The whole data set is available on demand to the corresponding author.

## Abbreviations

ED: Emergency department
OR: Odds ratio
CI: Confidence interval
CHF: Chronic Heart Failure
PAOD: Peripheral arterial occlusive disease
DVT: Deep venous thrombosis
COPD: Chronic Obstructive Pulmonary Disease
CKD: Chronic kidney disease
SST: Skin and soft tissues
GI: Gastrointestinal
SBP: Systolic Blood Pressure
DBP: Diastolic Blood Pressure
MBP: Mean Blood Pressure
GCS: Glasgow Coma Scale
SOFA: Sequential Organ Failure Assessment
qSOFA: “Quick” Sequential Organ Failure Assessment
ICU: Intensive Care Unit
