# Correction to: Septic patients without obvious signs of infection at baseline are more likely to die in the ICU

**DOI:** 10.1186/s12879-022-07255-z

**Published:** 2022-03-16

**Authors:** Francesco Campanelli, Agnès Soudry-Faure, Aurélie Avondo, Jean-Baptiste Roudaut, Jean-Pierre Quenot, Patrick Ray, Pierre-Emmanuel Charles

**Affiliations:** 1 Centre Régional Universitaire Des Urgences, Hôpital F. Mitterrand, C.H.U. DIJON, Bd Mal de Lattre de Tassigny, Dijon, France; 2 Centre d’Investigation Clinique, Hôpital F. Mitterrand, C.H.U. Dijon, 14 rue Gafarel, Dijon, France; 3 Service de Médecine Intensive Réanimation, Hôpital F. Mitterrand, C.H.U. DIJON, 14 rue Gafarel, B.P. 7790821079 Dijon Cedex, France; 4 Laboratoire Lipness, U.M.R. 1231, INSERM, Université de Bourgogne-Franche Comté, 7 Bd Jeanne d’Arc, Dijon, France; 5 INSERM, CIC 1432, Module Épidémiologie Clinique, Université de Bourgogne-Franche Comté, Dijon, France

## Correction to: BMC Infectious Diseases (2022) 22:205 10.1186/s12879-022-07210-y

Following publication of the original article [1], the authors identified that the given name and family name were erroneously transposed.

The incorrect authors’ names list is:

Campanelli Francesco^1^, Soudry-Faure Agnès^2^, Avondo Aurélie^1^, Roudaut Jean-Baptiste^3^, Quenot Jean-Pierre^3,4,5^, Ray Patrick^1^ and Charles Pierre-Emmanuel^3,4^*

The correct authors’ names list is:

Francesco Campanelli^1^, Agnès Soudry-Faure^2^, Aurélie Avondo^1^, Jean-Baptiste Roudaut^3^, Jean-Pierre Quenot^3,4,5^, Patrick Ray^1^ and Pierre-Emmanuel Charles^3,4^*

The author group has been updated above and the original article [1] has been corrected.
