# Supplementary material for: Septic patients without obvious signs of infection at baseline are more likely to die in the ICU
Source: BMC Infect Dis. 2022 Mar 2;22:205. doi: 10.1186/s12879-022-07210-y (PMC8889780; doi:10.1186/s12879-022-07210-y)
Supplement: Supplementary file 1 — Additional file 1: Figure S1. 28-day cumulative survival according to the sepsis presentation (i.e., Implicit vs. Vague) at the emergency department in 348 critically ill patients diagnosed with sepsis. [file 12879_2022_7210_MOESM1_ESM.docx]

**Figure S1 :** 28-day cumulative survival according to the sepsis presentation (i.e., Implicit *vs*. Vague) at the emergency department in 348 critically ill patients diagnosed with sepsis.

**
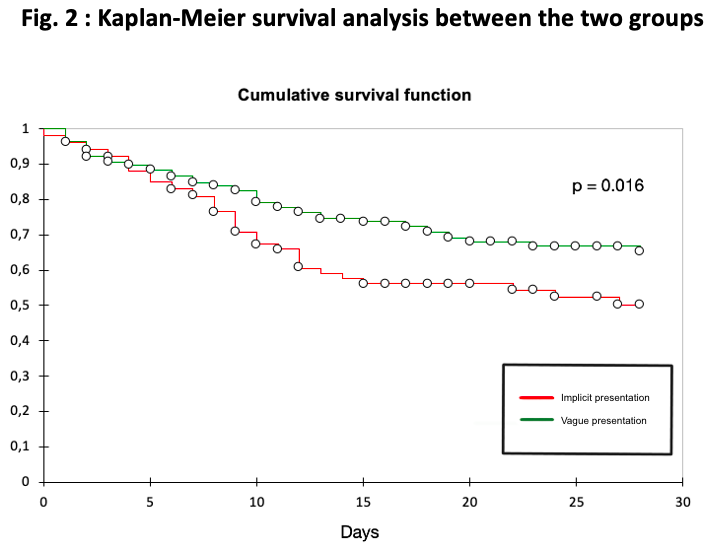
**
