# Supplementary material for: Septic patients without obvious signs of infection at baseline are more likely to die in the ICU
Source: BMC Infect Dis. 2022 Mar 2;22:205. doi: 10.1186/s12879-022-07210-y (PMC8889780; doi:10.1186/s12879-022-07210-y)
Supplement: Supplementary file 2 — Additional file 2: Figure S2. Probability of 28-day death according to Body Temperature (BT) measured at the ED in 348 ICU patients diagnosed with sepsis. [file 12879_2022_7210_MOESM2_ESM.docx]

**
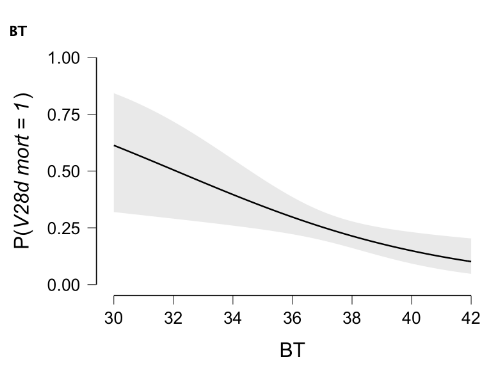
Figure S2 : Probability of 28-day death according to Body Temperature (BT) measured at the ED in 348 ICU patients diagnosed with sepsis.**
