# Supplementary material for: Septic patients without obvious signs of infection at baseline are more likely to die in the ICU
Source: BMC Infect Dis. 2022 Mar 2;22:205. doi: 10.1186/s12879-022-07210-y (PMC8889780; doi:10.1186/s12879-022-07210-y)
Supplement: Supplementary file 3 — Additional file 3: Table S1. Severity criteria of infection according to the clinical presentation of sepsis at the emergency department. [file 12879_2022_7210_MOESM3_ESM.docx]

**Table S1: Severity criteria of infection according to the clinical presentation of sepsis at the emergency department.**

SBP: Systolic Blood Pressure, DBP: Diastolic Blood Pressure, MBP: Mean Blood Pressure, GCS: Glasgow Coma Scale, SOFA: Sequential Organ Failure Assessment

| Total, n = 348 | Explicit  n = 245 | Vague  n = 103 | *p* |
| --- | --- | --- | --- |
| SBP, mean mmHg (SD) | 111 (31) | 132 (29) | < .001 |
| DBP, mean mmHg (SD) | 63 (18) | 73 (17) | < .001 |
| MBP, mean mmHg (SD) | 79 (21) | 93 (19) | < .001 |
| Hearth Rate, mean (SD) | 101 (28) | 98 (27) | 0.18 |
| SpO2, mean % (SD) | 93 (7) | 93 (7) | 0.57 |
| Respiratory Rate, mean (SD) | 27 (8) | 30 (12) | 0.26 |
| GCS, median (IQR) | 14 (1) | 14 (0) | 0.27 |
| Body Temperature, mean °C (SD) | 37.6 (1.9) | 37.1 (0.7) | < .001 |
| qSOFA, mean (SD) | 1.3 (0.8) | 0.8 (0.8) | < .001 |
| SOFA at sepsis diagnosis, mean (SD) | 5.2 (3.1) | 4.7 (3.2) | 0.09 |
