# Supplementary material for: Septic patients without obvious signs of infection at baseline are more likely to die in the ICU
Source: BMC Infect Dis. 2022 Mar 2;22:205. doi: 10.1186/s12879-022-07210-y (PMC8889780; doi:10.1186/s12879-022-07210-y)
Supplement: Supplementary file 4 — Additional file 4: Table S2. Confirmed pathogen according to the clinical presentation of sepsis at the emergency department. [file 12879_2022_7210_MOESM4_ESM.docx]

**Table S2: Confirmed pathogen according to the clinical presentation of sepsis at the emergency department.**

| Pathogen | Explicit  n = 245 | Vague  n = 103 | *p* |
| --- | --- | --- | --- |
| S. aureus (%) | 15 (6.1) | 8 (7.8) | 0.57 |
| S. pneumoniae (%) | 17 (6.9) | 3 (2.9) | 0.14 |
| E. coli (%) | 55 (22.4) | 11 (10.7) | 0.013 |
| K. pneumoniae (%) | 6 (2.4) | 9 (8.7) | 0.09 |
| P. aeruginosa (%) | 10 (4.1) | 5 (4.4) | 0.61 |
