# Supplementary material for: Septic patients without obvious signs of infection at baseline are more likely to die in the ICU
Source: BMC Infect Dis. 2022 Mar 2;22:205. doi: 10.1186/s12879-022-07210-y (PMC8889780; doi:10.1186/s12879-022-07210-y)
Supplement: Supplementary file 5 — Additional file 5: Table S3. Clinical predictors of all cause death at day-28. [file 12879_2022_7210_MOESM5_ESM.docx]

**Table S3: Clinical predictors of all cause death at day-28.**

OR = Odds Ratio, CI = Confidence Interval, CHF = Chronic Heart Failure, PAOD = Peripheral Arterial Occlusive Disease, DVT = Deep Venous Thrombosis, COPD = Chronic Obstructive Pulmonary Disease, CKD = Chronic Kidney Disease, ED = Emergency Department, SBP = Systolic Blood Pressure, DBP = Diastolic Blood Pressure, MBP = Mean Blood Pressure, GCS = Glasgow Coma Scale, SOFA = Sequential Organ Failure Assessment, SST = Skin and Soft Tissues, GI = Gastrointestinal

| Total, n = 348 | 28-day survivors  n = 240 | | | | | | | 28-day non-survivors  n = 108 | | | | OR | | | | 95% CI | | | | | *p* | | | | |
| --- | --- | --- | --- | --- | --- | --- | --- | --- | --- | --- | --- | --- | --- | --- | --- | --- | --- | --- | --- | --- | --- | --- | --- | --- | --- |
| Age, median year (IQR) | 70 (50-90) | | | | | | | 79 (61-97) | | | | 1.04 | | | | 1.02-1.06 | | | | | <.001 | | | | |
| Gender female (%) | 85 (35.4) | | | | | | | 45 (41.7) | | | | 1.30 | | | | 0.82-2.07 | | | | | 0.26 | | | | |
| Smoke (%) | 85 (35.4) | | | | | | | 24 (22.2) | | | | 0.52 | | | | 0.31-0.88 | | | | | 0.014 | | | | |
| Alcohol (%) | 56 (23.3) | | | | | | | 19 (17.6) | | | | 0.70 | | | | 0.39-1.25 | | | | | 0.23 | | | | |
| Arterial Hypertension (%) | 114 (47.5) | | | | | | | 66 (61.1) | | | | 1.74 | | | | 1.09-2.76 | | | | | 0.019 | | | | |
| Chronic Heart Failure (%) | 44 (18.3) | | | | | | | 22 (20.3) | | | | 1.14 | | | | 0.64-2.02 | | | | | 0.65 | | | | |
| Myocardial Infarction (%) | 51 (21.3) | | | | | | | 22 (20.3) | | | | 0.95 | | | | 0.54-1.66 | | | | | 0.85 | | | | |
| Atrial Fibrillation (%) | 70 (29.2) | | | | | | | 42 (38.9) | | | | 1.54 | | | | 0.96-2.49 | | | | | 0.072 | | | | |
| PAOD (%) | 36 (15) | | | | | | | 21 (19.4) | | | | 1.37 | | | | 0.75-2.48 | | | | | 0.30 | | | | |
| DVT (%) | 29 (12.1) | | | | | | | 8 (7.4) | | | | 0.58 | | | | 0.26-0-32 | | | | | 0.19 | | | | |
| Diabetes Mellitus (%) | 70 (29.2) | | | | | | | 35 (32.4) | | | | 1.16 | | | | 0.71-1.90 | | | | | 0.54 | | | | |
| COPD (%) | 33 (13.8) | | | | | | | 19 (17.6) | | | | 1.34 | | | | 0.72-2.48 | | | | | 0.35 | | | | |
| Asthma (%) | 15 (6.3) | | | | | | | 3 (2.8) | | | | 0.43 | | | | 0.12-1.51 | | | | | 0.18 | | | | |
| CKD (%) | 34 (14.2) | | | | | | | 21 (19.4) | | | | 1.46 | | | | 0.80-2.66 | | | | | 0.21 | | | | |
| Stroke (%) | 21 (8.8) | | | | | | | 12 (11.1) | | | | 1.30 | | | | 0.61-2.76 | | | | | 0.49 | | | | |
| Cirrhosis (%) | 15 (6.3) | | | | | | | 9 (8.3) | | | | 1.36 | | | | 0.58-3.22 | | | | | 0.48 | | | | |
| Immunosuppressive Drugs (%) | 39 (16.3) | | | | | | | 16 (14.8) | | | | 0.89 | | | | 0.48-1.67 | | | | | 0.73 | | | | |
| Haematological malignancies (%) | 32 (13.3) | | | | | | | 12 (11.1) | | | | 0.81 | | | | 0.40-1.65 | | | | | 0.56 | | | | |
| Solid cancer (%) | 58 (24.2) | | | | | | | 23 (21.3) | | | | 0.85 | | | | 0.49-1.47 | | | | | 0.56 | | | | |
| ED diagnosis (%) | 196 (81.7) | | | | | 79 (73.1) | | | | | | | | 0.61 | 0.36-1.05 | | | | | | | 0.07 | | |  |
| BT ≥ 38.5°C (%) | 87 (36.3) | | | | | 19 (17.6) | | | | | | | | 0.37 | 0.21-0.66 | | | | | | | <.001 | | |  |
| BT < 36°C (%) | 25 (10.4) | | | | | 21 (19.4) | | | | | | | | 2.08 | 1.10-3.90 | | | | | | | 0.02 | | |  |
| SBP ≤ 90 mmHg (%) | 56 (23.3) | | | | | 18 (16.7) | | | | | | | | 0.66 | 0.36-1.18 | | | | | | | 0.16 | | |  |
| MBP ≤ 65 mmHg (%) | 59 (24.6) | | | | | 17 (15.7) | | | | | | | | 0.57 | 0.31-1.04 | | | | | | | 0.065 | | |  |
| Productive cough (%) | 34 (14.2) | | | | | 8 (7.4) | | | | | | | | 0.48 | 0.22-1.09 | | | | | | | 0.07 | | |  |
| Dysuria (%) | 6 (2.5) | | | | | 1 (0.9) | | | | | | | | 0.36 | 0.04-3.06 | | | | | | | 0.33 | | |  |
| Skin Redness (%) | 8 (3.3) | | | | | 3 (2.8) | | | | | | | | 0.83 | 0.21-3.19 | | | | | | | 0.78 | | |  |
| Referral for Infection (%) | 50 (20.8) | | | | | 12 (11.1) | | | | | | | | 0.47 | 0.24-0.93 | | | | | | | 0.03 | | |  |
| Arrival SBP, mean mmHg (SD) | | 116 (31) | | 120 (32) | | | | | | | 1.00 | | | | | | 0.99-1.01 | | | 0.19 | | | |  |  |
| Arrival DBP, mean mmHg (SD) | | 66 (19) | | 66 (17) | | | | | | | 1.00 | | | | | | 0.99-1.01 | | | 0.94 | | | |  |  |
| Arrival MBP, mean mmHg (SD) | | 82 (22) | | 84 (20) | | | | | | | 1.00 | | | | | | 0.99-1.01 | | | 0.49 | | | |  |  |
| Arrival Hearth Rate, mean (SD) | | 102 (27) | | 95 (30) | | | | | | | 0.99 | | | | | | 0.98-0.99 | | | 0.016 | | | |  |  |
| Arrival SpO2, mean % (SD) | | 93 (8) | | 93 (7) | | | | | | | 1.00 | | | | | | 0.97-1.04 | | | 0.97 | | | |  |  |
| Arrival GCS, median (IQR) | | 15 (15-15) | | 15 (15-15) | | | | | | | 0.89 | | | | | | 0.81-0.99 | | | 0.048 | | | |  |  |
| Arrival Body Temperature,  mean °C (SD) | | 37.7 (1.5) | | 36.9 (1.7) | | | | | | | 0.75 | | | | | | 0.65-0.87 | | | <.001 | | | |  |  |
| Arrival qSOFA, mean (SD) | | | 1.1 (0.8) | | 1.2 (0.8) | | | | | | | | 1.10 | | | 0.83-1.46 | | | | | | | 0.48 |  |  |
| Pulmonary source of infection (%) | | | 116 (48.3) | | | | 53 (49.1) | | | 1.03 | | | | | | | | 0.65-1.62 | 0.89 | | | | |  |  |
| Urinary source of infection (%) | | | 51 (21.3) | | | | 19 (17.6) | | | 0.78 | | | | | | | | 0.43-1.41 | 0.41 | | | | |  |  |
| SST source of infection (%) | | | 12 (5) | | | | 8 (7.4) | | | 1.52 | | | | | | | | 0.60-3.83 | 0.38 | | | | |  |  |
| GI source of infection (%) | | | 15 (6.3) | | | | 5 (4.6) | | | 0.73 | | | | | | | | 0.26-2.06 | 0.55 | | | | |  |  |
| Biliary source of infection (%) | | | 12 (5) | | | | 5 (4.6) | | | 0.92 | | | | | | | | 0.32-2.69 | 0.88 | | | | |  |  |
| Implicit presentation (%) | | | 61 (25.4) | | | | 42 (38.9) | | 1.87 | | | | | | | | | 1.15-3.03 | 0.011 | | | | |  |  |
| Identification of Infection Site (%) | | | 231 (96.3) | | | | 100 (92.6) | | 0.49 | | | | | | | | | 0.18-1.29 | 0.14 | | | | |  |  |
| Bacteremia (%) | | | 80 (33.3) | | | | 37 (34.3) | | 1.04 | | | | | | | | | 0.64-1.69 | 0.87 | | | | |  |  |
| Time to diagnosis, mean hours (SD) | | | 8 (21) | | | | 8.5 (16) | | 1.00 | | | | | | | | | 1.00-1.00 | 0.79 | | | | |  |  |
| Time to treatment, mean hours (SD) | | | 10 (27) | | | | 10 (17) | | 1.00 | | | | | | | | | 1.00-1.00 | 0.79 | | | | |  |  |
| Time to ICU, mean hours (SD) | | | 40 (113) | | | | 32 (89) | | 1.00 | | | | | | | | | 1.00-1.00 | 0.57 | | | | |  |  |
| ICU direct admission (%) | | | 167 (69.6) | | | | 76 (67.6) | | 0.91 | | | | | | | | | 0.56-1.49 | 0.71 | | | | |  |  |
| Overall SOFA, mean (SD) | | | 4.5 (2.7) | | | | 6.3 (3.6) | | 1.20 | | | | | | | | | 1.12-1.29 | <.001 | | | | |  |  |
| ABT Adequacy (%) | | | 121 (50.4) | | | | 56 (51.8) | | 0.97 | | | | | | | | | 0.57-1.66 | 0.61 | | | | |  |  |
